# Effects of Gamma-Tocotrienol on Partial-Body Irradiation-Induced Intestinal Injury in a Nonhuman Primate Model

**DOI:** 10.3390/antiox11101895

**Published:** 2022-09-25

**Authors:** Sarita Garg, Tarun K. Garg, Isabelle R. Miousse, Stephen Y. Wise, Oluseyi O. Fatanmi, Alena V. Savenka, Alexei G. Basnakian, Vijay K. Singh, Martin Hauer-Jensen

**Affiliations:** 1 Division of Radiation Health, Department of Pharmaceutical Sciences, University of Arkansas for Medical Sciences, Little Rock, AR 72205, USA; 2 Department of Biochemistry and Molecular Biology, University of Arkansas for Medical Sciences, Little Rock, AR 72205, USA; 3 UAMS Myeloma Center, University of Arkansas for Medical Sciences, Little Rock, AR 72205, USA; 4 Division of Radioprotectants, Department of Pharmacology and Molecular Therapeutics, F. Edward Hébert School of Medicine, Uniformed Services University of the Health Sciences, Bethesda, MD 20814, USA; 5 Armed Forces Radiobiology Research Institute, Uniformed Services University of the Health Sciences, Bethesda, MD 20814, USA; 6 Department of Pharmacology and Toxicology, University of Arkansas for Medical Sciences, Little Rock, AR 72205, USA; 7 John L. McClellan Memorial VA Hospital, Central Arkansas Veterans Healthcare System, Little Rock, AR 72205, USA

**Keywords:** gamma-tocotrienol, intestine, partial-body irradiation, nonhuman primates, radiation countermeasure

## Abstract

Exposure to high doses of radiation, accidental or therapeutic, often results in gastrointestinal (GI) injury. To date, there are no therapies available to mitigate GI injury after radiation exposure. Gamma-tocotrienol (GT3) is a promising radioprotector under investigation in nonhuman primates (NHP). We have shown that GT3 has radioprotective function in intestinal epithelial and crypt cells in NHPs exposed to 12 Gy total-body irradiation (TBI). Here, we determined GT3 potential in accelerating the GI recovery in partial-body irradiated (PBI) NHPs using X-rays, sparing 5% bone marrow. Sixteen rhesus macaques were treated with either vehicle or GT3 24 h prior to 12 Gy PBI. Structural injuries and crypt survival were examined in proximal jejunum on days 4 and 7. Plasma citrulline was assessed using liquid chromatography–tandem mass spectrometry (LC-MS/MS). Crypt cell proliferation and apoptotic cell death were evaluated using Ki-67 and TUNEL staining. PBI significantly decreased mucosal surface area and reduced villous height. Interestingly, GT3 increased crypt survival and enhanced stem cell proliferation at day 4; however, the effects seemed to be minimized by day 7. GT3 did not ameliorate a radiation-induced decrease in citrulline levels. These data suggest that X-rays induce severe intestinal injury post-PBI and that GT3 has minimal radioprotective effect in this novel model.

## 1. Introduction

Total- and partial-body irradiation (TBI/PBI) following a radiological or nuclear disaster can result in the potentially lethal gastrointestinal acute radiation syndrome (GI-ARS) and hematopoietic ARS (H-ARS) in a short period [1,2]. Such radiation-induced injuries require appropriate diagnosis and treatment [3]. Typically, these accidental radiological and/or nuclear exposures are more than likely to be non-uniform by nature. The ARS progression mainly depends on the radiation dose absorbed, the severity of exposure, and its distribution within the body [4]. Moreover, not all organs are equally radiosensitive. The GI and the hematopoietic system are amongst the most radiosensitive organs in the body. In addition, exposure to radiation is also a serious concern in cancer patients receiving abdominal radiotherapy [5]. Thus, there is a paramount need for the development of non-toxic, safe, and effective radiation medical countermeasures (MCM) to protect radiation-induced GI injury. 

Studies have shown that GI damage is a critical determinant of radiation-induced lethality in humans [6]. Massive intestinal stem cell depletion occurs after high doses of radiation. This compromises mucosal integrity and impairs epithelial regeneration [7]. The classical GI injury is mainly associated with the tremendous shortening or “blunting” of intestinal villi, death of clonogenic crypt cells, tight junctions disruption, mucosal barrier breakdown, inflammation, decreased nutrient absorption, diarrhea leading to electrolyte and fluid imbalance, and intestinal bleeding or infection, which may culminate to sepsis and eventually death [8,9,10]. Many strategies have been developed; however, to date, there is no United States Food and Drug Administration (FDA)-approved MCM for GI-ARS [11]. The four FDA-approved products for ARS address only H-ARS [12,13,14,15]. 

Over the last few decades, several natural compounds have been developed for the prevention and treatment of radiation-induced injury and compared to their synthetic analogs in clinical studies [16]. Among them, vitamin E is a potent antioxidant that regulates lipid peroxidation and controls free radical production within the body. The vitamin E family refers to a group of potent lipid-soluble, chain-breaking antioxidants, tocopherols (α, β, γ, δ), and tocotrienols (α, β, γ, δ), collectively known as tocols [17,18]. Both groups are differentiated by the methyl group position on the chromanol ring. Tocols are well known for their neuroprotective, anti-inflammatory, antioxidative, and radioprotective properties [16,19,20]. Their potent antioxidant activity helps protect cells from increased oxidative injury caused by free radical generation, one of the key mediators of radiation toxicity [17,18]. Importantly, tocotrienol is 1600 times more potent as an antioxidant than alpha-tocopherol [21] and is superior in protecting against oxidant injury and subsequent mitochondrial dysfunction [22]. Likewise, several studies report tocotrienols to be mechanistically stronger antioxidants than tocopherols [16,23]. GT3 is a well-known antioxidant and free radical scavenger [18,24] and has garnered significant attention in recent years. It has been reported to be one of the most promising prophylactic MCMs for H-ARS [24]. GT3, in addition to being a strong inhibitor of 3-hydroxy-3-methylglutaryl-coenzyme A (HMG-CoA) reductase [25,26], has also been shown to protect GI, vascular, and hematopoietic systems against radiation injury [27,28]. GT3 is currently under advanced development as a radioprotective MCM for use as pre-exposure prophylaxis for H-ARS [24]. Importantly, in a recent study, we showed that GT3 exhibited radioprotective function in intestinal epithelial and crypt cells in an NHP model exposed to a supralethal dose of 12 Gy TBI using gamma radiation [29]. 

In this study, we assessed the potential of GT3 to accelerate GI recovery in NHPs exposed to a radiation dose of 12 Gy PBI using X-rays, sparing 5% of bone marrow (BM). The NHP model with minimal sparing of 2.5% or 5% BM was developed to resemble accidental or deliberate radiation exposures in humans [30,31,32]. The goal of such exposures would likely allow performing side-by-side analysis of short- and long-term injury to organ systems, sparing the BM, in a dose- and time-dependent manner [30]. Further, as per the FDA Animal Rule, this model is utilized to study MCM for GI-ARS. [33,34,35,36]. Several studies have characterized this PBI model in depth, including the analysis of resulting tissue injury and tissue-plasma correlations of potential biomarkers, as well as its use for MCM development [30,31,35,37,38,39]. However, to our knowledge, this is the first report with GT3 using the X-ray partial-body NHP exposure model with LINAC. Results from this study demonstrated that a PBI dose of 12 Gy resulted in significant intestinal injury, including shortening of the villi and decreased mucosal surface area. GT3 treatment increased both crypt survival and stem cell proliferation at day 4; however, the effects did not persist by day 7. GT3 did not ameliorate a radiation-induced decrease in plasma citrulline levels. Taken together, these data suggest that X-rays induce substantial intestinal injury post-PBI at an early time point and that GT3 is minimally protective in the PBI model. 

## 2. Materials and Methods

### 2.1. Animals 

Sixteen naïve rhesus macaques (*Macaca mulatta*, Chinese sub-strain, 8 males and 8 females) were included in this study. The animals were between 3.6 and 5.5 years of age with a body weight between 5.45 and 10.35 kg. The animals were randomly divided into two groups so that half received GT3 (37.5 mg/kg, subcutaneously (sc)) and the other half received vehicle. The animals were procured from an approved NHP vendor (PrimGen, Hines, IL, USA). All animals were housed in a facility accredited by the Association for Assessment and Accreditation of Laboratory Animal Care (AAALAC)-International. Additional animal details and their housing have been described earlier [29]. All procedures involving animals were approved by the Institutional Animal Care and Use Committee (BIOQUAL Inc. Rockville, MD, USA, Protocol #18-060) and the Department of Defense Animal Care and Use Review Office (ACURO). This protocol was approved by BIOQUAL IACUC on 27 June 2018 and ACURO on 8 March 2019. The study was conducted in strict accordance with the recommendations in the Guide for the Care and Use of Laboratory Animals. 

### 2.2. Experimental Design and Irradiation

The purpose of this study was to evaluate the efficacy of a specific MCM, GT3, against intestinal injury in a GI-ARS model exposed to PBI. The various analyses helped with understanding the mechanism of action of this particular MCM in rescuing the GI tract of irradiated NHPs. A radiation dose of 12 Gy PBI was used (5% sparing of bone marrow—hind limbs fibula, tibia, and feet not irradiated—in a LINAC where shielding was not required for sparing). The 16 NHPs were further divided into two groups (eight NHPs each), where one group received 37.5 mg/kg of GT3 and the other received the corresponding vehicle sc in the dorsal scapular region 24 h prior to radiation exposure (Table 1). Blood was collected at intervals, and the animals were euthanized according to a schedule for tissue collection on various days post-irradiation. The samples were analyzed for histopathology and various biomarkers. 

### 2.3. Partial-Body Irradiation

The fasting, transportation, and sedation of the NHPs were as described earlier for TBI [29]. For PBI, NHPs were irradiated one at a time using a 4 MV photon beam from an Elekta Infinity clinical LINAC. Anterior/posterior (AP) measurements of the NHPs at the location of the absorbed dose target (“core of the abdomen”) were obtained with a digital caliper. Irradiation procedures with LINAC and dosimetry have been described previously [40]. 

Our study plan was to harvest 3 animals each on days 4 and 7 and two animals on day 10 post-irradiation. During execution of the study, all remaining animals for days 7 and 10 became moribund by day 7. Hence, following IACUC guidelines, all 5 animals in each group were harvested on day 7.

### 2.4. Drug Preparation and Administration

GT3 and vehicle were supplied by Callion Pharma (Jonesborough, TN) as an injectable solution at 50 mg/mL. Immediately before administration, the GT3 and vehicle solutions were thoroughly mixed using a lab vortexer. GT3 or vehicle were administered *sc* at a dose of 37.5 mg/kg at 24 h prior to irradiation (the exact volume administered to each NHP depended on the body weight). Injections were performed using a sterile 21–25-gauge needle with a length of ¾-1”. The site of injection (dorsal scapular region (midline)) was prepared as a surgical site: hair was removed with a #40 surgical clipper blade. The site was disinfected to reduce any adverse skin reactions, rash/eruption, inflammation, or irritation [29].

### 2.5. Blood and Intestinal Tissue Collection

Three NHPs from each group were euthanized on day 4, and five NHPs were euthanized on day 7 post-irradiation (see Table 2). Details of euthanasia and tissue collection are discussed in earlier publication [29]. Blood samples were collected on day 7 (pre-irradiation) and days 1, 4, and 7 for citrulline analysis. Tubes containing K3-EDTA as anti-coagulant were used to collect blood by venipuncture and were centrifuged at 1500 RCF at 4 °C for 10 min. Plasma was collected and stored at −80 °C until further analysis. 

### 2.6. Assessment of PBI-Induced Change in Plasma Citrulline Levels 

Citrulline is a minimally invasive and well-studied biomarker for functional enterocyte mass [38,41,42]. Studies have shown a significant negative correlation between circulating citrulline levels and the radiation dose [42,43,44]. Here, we collected blood plasma for citrulline analysis at day 7, day 1, day 4, and day 7 post-irradiation. 

Citrulline concentrations were assessed using the high throughput liquid chromatography–tandem mass spectrometry (LC-MS/MS) methodology previously described by our group [45]. Briefly, calibration standards, blanks, and QCs were prepared using a surrogate matrix (1% BSA in PBS) because citrulline is endogenous in mouse and NHP plasma. In total, 1% BSA in PBS (10 µL) was added to 0.5 mL of acetonitrile/water/formic acid (85/14.8/0.2). Citrulline (10 µL) was added to achieve concentrations ranging from 0.125–100 µM. Samples were prepared by adding 10 µL of plasma to 0.5 mL of acetonitrile/water/formic acid (85/14.8/0.2). Internal standard (citrulline-d6, 10 µL) was added to each standard and sample, and they were vortexed and then centrifuged for 5 min at 21,000× *g*. The supernatant was transferred to an autosampler vial and analyzed by LC/MS/MS. 

The LC/MS/MS system consisted of Shimadzu system (Columbia MD) equipped with LC20-AD dual HLPC pumps, an SIL20-AC HT autosampler, and a DGU-20A2 in-line degasser. Detection was performed using an Applied BioSystems 4000 QTRAP (Applied Biosystems, Foster City, CA, USA) triple quadrupole mass spectrometer operated in the positive ion mode. Mass calibration and data acquisition and quantitation were performed using Applied Biosystem Analyst 1.6.2 software (Applied Biosystems, Foster City, CA, USA). 

Separation of the citrulline and the internal standards from the sample matrix was achieved using a Phenomenex Kinetix HILIC, 50 × 2.1 mm 2.6 µm particle column kept at ambient temperature. The mobile phase was delivered at a flow rate of 400 µL/min using a gradient elution profile consisting of DI water with 0.1% formic acid, 0.2% acetic acid and 0.005% TFA (A) and acetonitrile with 0.1% formic acid, and 0.2% acetic acid and 0.005% TFA (B). Mobile phase B, which began at 95% and decreased to 85% over 1.5 min and then decreased to 70% over 0.5 min, was held at 70% for 1 min and then returned to 95% over 0.5 min and equilibrated for 4.5 min. The sample injection volume was 2 µL. The analyte and internal standard were detected using multiple reaction monitoring (MRM) for the following transitions: citrulline (*m*/*z* 176.2 → 159), enalaprat (*m*/*z* 182.2 → 165). 

### 2.7. Assessment of PBI-Induced Histological and Morphometric Injury

The putative role of GT3 in combating radiation-induced intestinal injury was assessed by examining histological changes within the proximal jejunum. As described earlier, mucosal surface length, villous height, and crypt depth were measured on jejunum tissue sections stained with H&E by using a computer-assisted image analysis platform [29].

#### 2.7.1. Mucosal Surface Length 

We have previously measured mucosal surface area in mouse and rat intestines with a stereological projection/cycloid method [46,47]. Due to structural differences in the intestine of NHPs (NHPs have plicae circulares, whereas mice and rats do not), we used computer-assisted Image-Pro^®^ Premier software (Meyer Instruments, Houston, TX, USA) to measure the length of intestinal mucosal surface area in the NHP model [29]. All measurements were performed at 4× magnification in six to eight areas of intestinal segments. The average of the measurements from those 6–8 areas were used as a single value for statistical analyses. 

#### 2.7.2. Mucosal Villus Height and Crypt Depth 

Assessments of mucosal villus height and crypt depth were obtained with computer-assisted Image-Pro Premier software. Measurements of villus height were carried out on images captured at 4× magnification in six to eight areas of intestinal segments, while crypt depth measurements were performed on images captured at 10× in eight to ten areas. As described previously, mucosal villus height was measured from the tip to the base of each villus, while crypt depth was measured from the crypt base to the tip opening [29]. In each sample, the average of the measurements was used as a single value for statistical analyses. 

### 2.8. Intestinal Crypt Colony Assay

The microcolony crypt cell survival assays have been described in detail elsewhere [29,48,49]. Surviving and regenerating crypts were counted in five H&E-stained transverse sections of the proximal jejunum on days 4 and 7 post-PBI. H&E slides were scanned at 20× in an Aperio CS2 scanner (Leica Biosystems, Deer Park, IL, USA) and analyzed with Imagescope software (Aperio Technologies). Surviving crypts containing ≥10 adjacent, chromophilic, non-Paneth cells were counted in five circumferences of proximal jejunum per animal, and microcolony survival was expressed as the average number of surviving crypts per circumference. The average from each animal was considered a single value for statistical purposes. 

### 2.9. TUNEL Assay

TUNEL assays were performed as described [29,50]. The assay was performed on jejunum sections obtained at day 4 and day 7 post-PBI using an In Situ Cell Death Detection Kit (Roche Diagnostics, Indianapolis, IN, USA) according to the manufacturer’s instructions. Jejunum sections were incubated with a mixture of terminal deoxynucleotidyl transferase (TdT) and fluorescein (FITC)-labeled precursor in cacodylate-based buffer for 1 h at 37 °C. The slides were rinsed three times in 0.05% Tween-20 in PBS and were then counterstained with 4′,6-diamidino-2-phenylindole (DAPI, Invitrogen, Carlsbad, CA, USA) to visualize cell nuclei. They were then mounted with Prolong Gold Antifade Mountant (Invitrogen, Carlsbad, CA, USA). TUNEL specificity was ascertained by substituting the mixture of TdT and probe with the cacodylate buffer. The green spectrum (FITC) and blue spectrum (DAPI) were used to detect TUNEL-positive cells and nuclei, respectively. Image acquisition and analysis were performed as described earlier [29]. Issues with regard to TUNEL image identification were resolved according to the classification of TUNEL images described in a recent review [51]. 

### 2.10. Fluorescent Microscopy 

Immunostaining of NHP jejunum sections was performed as described elsewhere [29,52]. Deparaffinated sections from the jejunum were rehydrated and then incubated with rabbit anti-claudin-2 (1:100), rabbit anti-Occludin (1:500), rabbit anti-β-Catenin (1:2000) (all from cell signaling technology, Danvers, MA, USA), rat anti-Ki-67 (1:200), mouse anti-Zona-1(1:450), mouse anti-claudin-7 (1:800), and mouse anti-E-Cadherin (1:2500) (Invitrogen, Carlsbad, CA, USA) in blocking buffer. The primary antibodies were detected using donkey anti-rabbit/rat secondary IgG-AlexaFluor 594 conjugates/donkey anti-mouse Alexafluor 488 conjugate/goat anti-rabbit Alexafluor 488 conjugate/goat anti-mouse Alexafluor 594 conjugate (1:800, Invitrogen, Carlsbad, CA, USA). The sections were then counterstained with 4′,6-diamidino-2-phenylindole (DAPI, Thermo Fisher, Waltham, MA, USA) to visualize cell nuclei and mounted with Prolong^®^ Gold Antifade Mountant (Invitrogen, Carlsbad, CA, USA). The images were captured and analyzed as described earlier [29]. 

### 2.11. Immunohistochemistry

Immunohistochemical staining was performed with conventional techniques using avidin–biotin complex, diaminobenzidine chromogen, and hematoxylin counterstaining [29]. Monoclonal mouse anti-proliferating cell nuclear antigen antibody (clone PC-10, EMD Millipore, Billerica, MA, USA) was used to detect proliferating cell nuclear antigen (PCNA) on proximal jejunum sections obtained on days 4 and 7 post-PBI. Sections were deparaffinized, rehydrated, and blocked for endogenous peroxidase, followed by incubation with mouse anti-PCNA (1:200, EMD Millipore) and then with the biotinylated goat anti-mouse IgG (1:400, Vector Laboratories, Newark, CA, USA). The sections were subsequently incubated with avidin–biotin–peroxidase complex (1:100, Vector Laboratories), and peroxidase binding was revealed using 0.5 mg/mL 3,3-diaminobenzidine tetrahydrochloride solution (Sigma-Aldrich, St. Louis, MO, USA). The slides were then rinsed and counterstained with Harris’s hematoxylin, dehydrated, and coverslipped. PCNA stained slides were scanned using the Aperio scanner CS2 at 20×.

### 2.12. Statistical Analysis 

The statistical analysis of all data was performed using the GraphPad Prism Version 9.1.0 (GraphPad Software, San Diego, CA, USA). Comparison of multiple means was performed with *ANOVA.* Pairwise comparisons were performed with Student’s *t*-test. The statistical significance level used was *p* < 0.05. 

## 3. Results

### 3.1. PBI-Induced Structural Injury in Proximal Jejunum 

**Figure 1:**
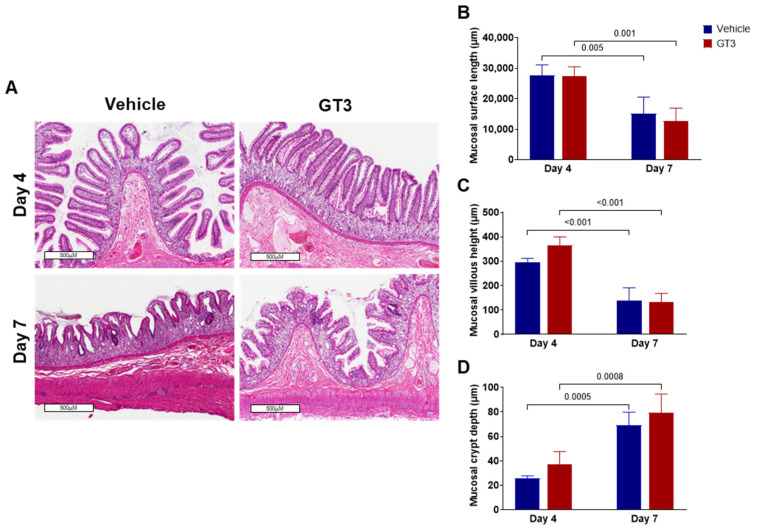
Effects of PBI on intestinal damage at day 4 and day 7. (**A**) Representative images showing the cross-sections of proximal jejunum of NHPs treated with vehicle and GT3 at days 4 and 7 post-PBI. Histogram showing the measurements of intestinal injury such as (**B**) mucosal surface length, (**C**) villous height, and (**D**) crypt depth. The data are presented as average ± SEM; *N* = 3 (day 4) and *N* = 5 (day 7).

High doses of radiation often result in shortening of the villi/villus blunting, which may be due to the loss of epithelial cells, thus compromising the mucosal integrity of the intestine. Here, the role of GT3 was determined in modulating irradiation-induced intestinal structural injury in the jejunum sections of GI at days 4 and 7 post-PBI. Radiation resulted in decreased mucosal surface length and shortening of the villi in both vehicle and GT3-treated groups by day 7, as shown in Figure 1A. Shrinkage in crypt size/crypt loss were noted at both day 4 and day 7.

Consistent with our findings in NHPs exposed to 12 Gy TBI [29], morphometric analysis revealed that PBI significantly reduced mucosal surface length at day 7 in both vehicle- (*p* < 0.005) and GT3-treated (*p* < 0.001) groups compared to their respective control groups on day 4. However, no difference was noted between vehicle- and GT3-treated groups when compared at either day 4 or day 7 (Figure 1B). Likewise, villous height was significantly reduced at day 7 in both vehicle- (*p* < 0.001) and GT3-treated groups (*p* < 0.001) when compared to their respective groups at day 4 (Figure 1C), but no significant difference was noted between the groups on either day. Based on published data, a strong positive correlation exists between survival of the crypt stem cell and crypt depth post-radiation [53]. Interestingly, when compared to day 4, crypt depth was found to be significantly increased at day 7 in both vehicle- (*p* < 0.0005) and GT3-treated groups (*p* < 0.0008), when compared to day 4 (Figure 1D), but no significant difference was observed between the groups on either day. 

### 3.2. Effects of PBI on Crypt Survival

**Figure 2:**
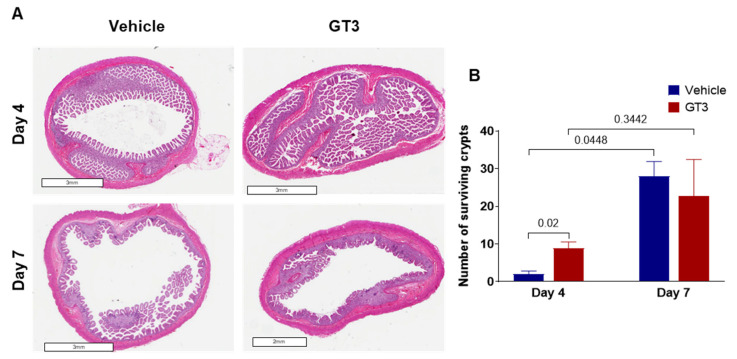
Effects of PBI on intestinal crypt survival. (**A**) Representative images showing the transverse sections of proximal jejunum from NHP treated with vehicle and GT3 at days 4 and 7 post-irradiation. (**B**) Surviving crypts. The data are presented as average ± SEM; *N* = 3 (day 4) and *N* = 5 (day 7).

Intestinal crypt survival was assessed at days 4 and 7 post-PBI (Figure 2A). Interestingly, GT3 significantly increased crypt survival at day 4 post-irradiation (*p* < 0.02); however, by day 7, no significant difference was noted between the vehicle- and GT3-treated groups (Figure 2B). In addition, a significant increase in crypt survival was observed at day 7 in the vehicle-treated group when compared to day 4 (*p* < 0.02) (Figure 2B). In contrast, GT3 did not influence crypt survival in NHPs of TBI with a supralethal dose of 12 Gy [29].

### 3.3. Effects of PBI on Plasma Citrulline Levels 

**Figure 3:**
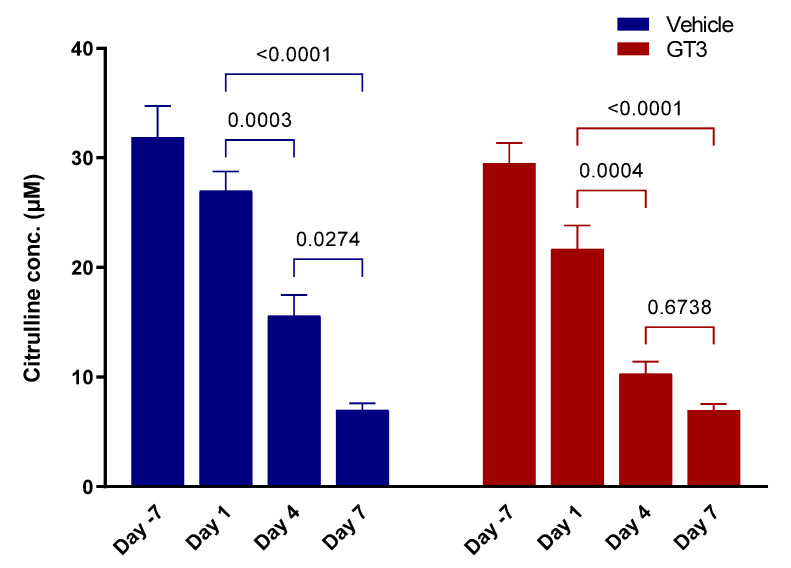
Effects of PBI on plasma citrulline levels. The levels of plasma citrulline were significantly decreased at all time points examined in both vehicle- and GT3-treated groups post-PBI. The data are presented as average ± SEM; *N* = 3 (day 4) and *N* = 5 (day 7).

Citrulline is a well-studied metabolite for radiation-induced GI damage [38,54,55]. Reduced plasma citrulline levels have been identified as one of the most sensitive, non-invasive biomarkers of injury in the intestine. Here, we demonstrate that PBI induced a time-dependent significant decrease in plasma citrulline levels in both vehicle- and GT3-treated groups, which persisted until day 7 (latest time point examined in this study). The drastic reduction in plasma citrulline levels was noted as early as day 4 post-irradiation (Figure 3). Plasma citrulline levels significantly decreased in both vehicle- and GT3-treated groups at day 4 (*p* < 0.0003; *p* < 0.0004) and day 7 (*p* < 0.0001) when compared to day 1, while a significant reduction was observed in the vehicle group only at day 7 when compared to day 4 (*p* < 0.02) (Figure 3). The basal plasma citrulline levels in vehicle- and GT3-treated groups at day—7 were 31.90 ± 2.84 µM and 29.50 ± 1.86 µM; however, the levels decreased progressively at days 1, 4, and 7 post-PBI with the vehicle group exhibiting citrulline concentration as 27.01 ± 1.76 µM, 15.63 ± 1.88 µM, and 7.03 ± 0.58 µM, while the GT3-treated group showed 21.70 ± 2.14 µM, 10.33 ± 1.08 µM, and 6.99 ± 0.55 µM levels, respectively. 

### 3.4. Effects of PBI on Cell Death in the Proximal Jejunum 

**Figure 4:**
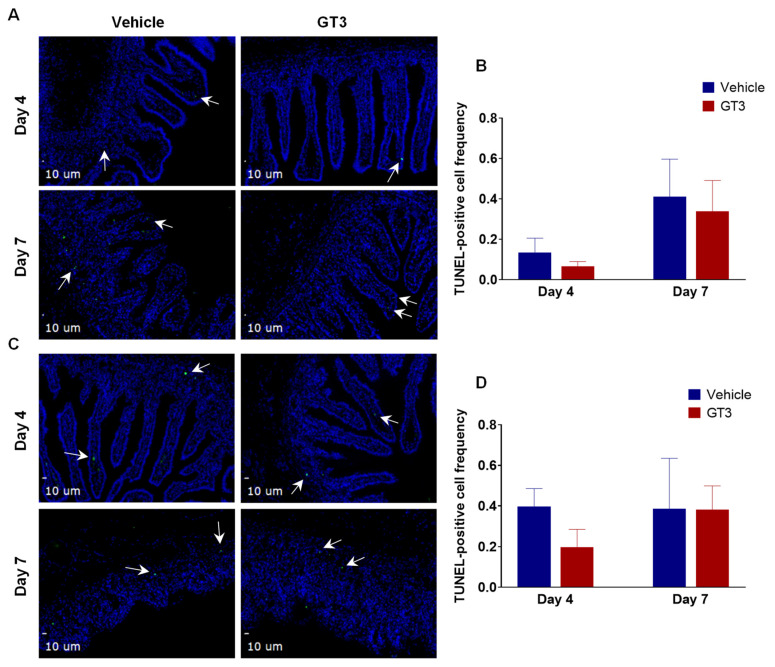
Effects of PBI on cell death in the proximal jejunum. (**A**) Representative photomicrograph of TUNEL-positive cells (green) in the jejunal villi. (**B**) Frequency of TUNEL-positive cells in villi at days 4 and 7 post-PBI. (**C**) Representative photomicrograph of TUNEL-positive cells (green) in the crypt. (**D**) Frequency of TUNEL-positive cells in the crypts at days 4 and 7 post-PBI. TUNEL-positive cells are shown by white arrows. The data are presented as average ± SEM; *N* = 3 (day 4) and *N* = 5 (day 7).

Radiation-induced cell death was assessed by TUNEL staining of NHP proximal jejunum sections at days 4 and 7 following PBI. The intestinal epithelium undergoes rapid renewal, which is dependent on the stem and progenitor cells that reside in the crypt [56]. Here, we observed no significant difference in the frequency of TUNEL-positive cells in the villi (Figure 4A,B) or crypts (Figure 4C,D) of vehicle- or GT3-treated groups at either day 4 or day 7. The data show that TUNEL positivity (irreversible cell death) was overall very low, no more than 0.4% of the total cells, and GT3 provided some mitigation of the injury at day 4 compared to the vehicle, which did not reach statistical significance. On the contrary, we noted a significant decrease in the frequency of TUNEL-positive cells in the villi of GT3-treated NHPs at both days 4 and 7 when exposed to a supralethal dose of 12 Gy TBI with gamma rays [29], thus suggesting different radiation qualities (gamma rays versus X-rays) may result in different biological effects. However, this could also be due to different mechanisms induced by TBI versus PBI and therefore differential effects of GT3 treatment, independent of the type of radiation used. Furthermore, the dose rate in the TBI study was 0.6 Gy/min while the dose rate in the PBI study was 1.4 Gy/min, and this may also be contributing to the different effects of GT3 in TBI versus PBI studies.

### 3.5. Effects of PBI on Cell Proliferation in the Jejunum Crypts 

The intestinal epithelial regeneration after radiation injury is crucial for re-establishment of the jejunum architecture [57,58,59]. This renewal is mainly dependent on the resident crypt cells that constantly serve to reconstitute the epithelial cells that are lost from the villous [59]. Ki-67, a nuclear antigen present in the proliferating cells, is a well-known and one of the most extensively used proliferation cell markers. Here, we assessed the effects of GT3 on the crypt cell proliferation at days 4 and 7 by determining Ki-67 expression in the jejunum exposed to 12 Gy partial-body radiation. 

**Figure 5:**
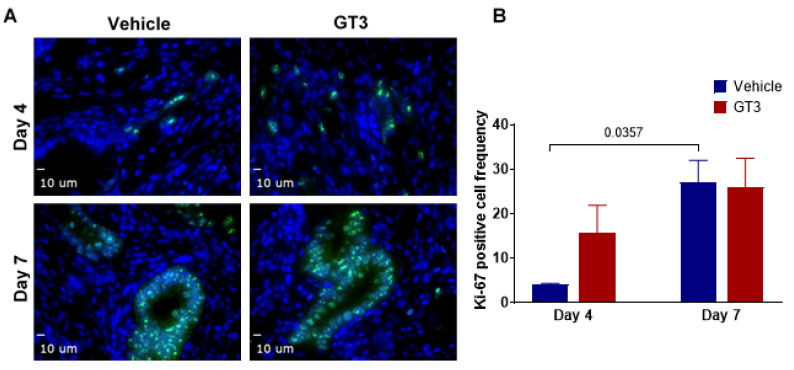
Effects of PBI on intestinal stem proliferation as assessed by Ki-67. (**A**) Representative photomicrograph of Ki-67-positive cells (green) in the crypts. (**B**) Mean fluorescence intensity of Ki-67 at days 4 and 7 post-PBI in vehicle- and GT3-treated groups. The data are presented as average ± SEM; *N* = 3 (day 4) and *N* = 5 (day 7).

**Figure 6:**
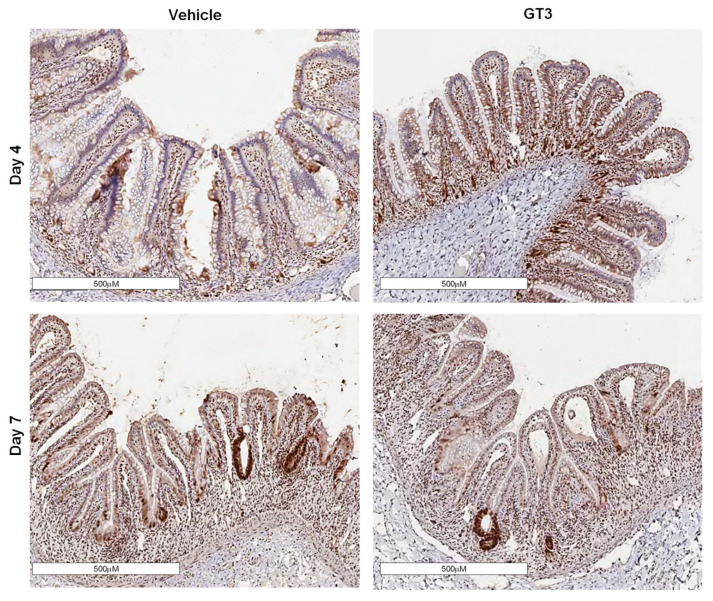
Effects of PBI on PCNA expression in proximal jejunum post-irradiation. Representative images showing the expression of PCNA in cross-sections of proximal jejunum of NHP treated with vehicle and GT3 prior to exposure to 12 Gy at day 4 and day 7 post-irradiation.

Interestingly, the GT3-treated group showed an increase in Ki-67-positive cells when compared to the vehicle at day 4, though the level of significance was not achieved (Figure 5A). On the contrary, the vehicle-treated group at day 7 demonstrated a significant increase in Ki-67-positive cells (*p* < 0.04) when compared to day 4 post-PBI (Figure 5B). However, no difference was noted between vehicle- and GT3-treated groups at day 7. This is in contrast to what we observed in NHPs exposed to 12 Gy TBI [29], where GT3 significantly increased Ki-67-positive cells at day 7 post-irradiation. In addition to Ki-67, PCNA expression, another critical marker for cell proliferation that plays an essential role in DNA synthesis/replication, was also evaluated. PCNA analysis also demonstrated an enhanced expression in proliferating crypts in GT3-treated groups at day 4, while no difference was observed between vehicle- and GT3-treated groups at day 7 (Figure 6). 

### 3.6. Effects of GT3 on PBI-Induced Alterations in Tight Junction-Related Proteins in the Jejunum

**Figure 7:**
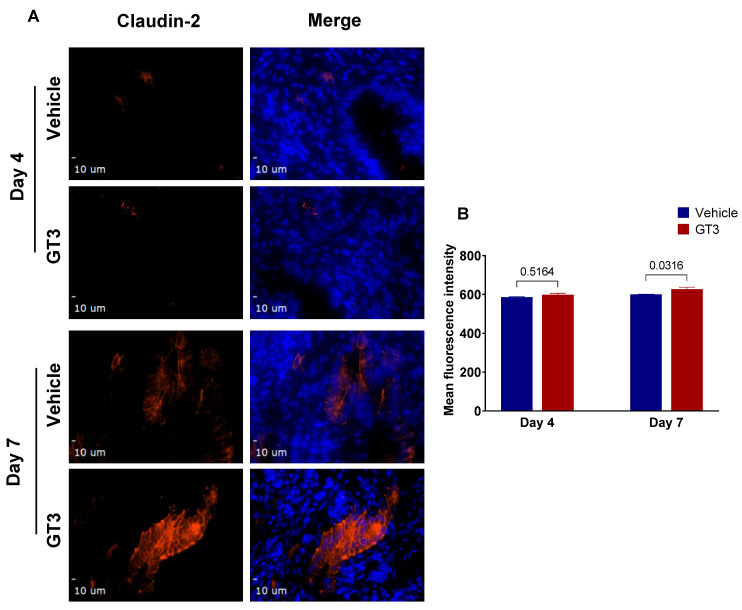
Effects of PBI on claudin-2 expression in the jejunum. (**A**) Representative photomicrograph of claudin-2 expression (red) in the cross-sections of the jejunum at day 4 and day 7 post-irradiation. (**B**) Quantitation of claudin-2 expression levels in jejunal crypts at time points indicated post-12 Gy PBI. The data are presented as average ± SEM; *N* = 3 (day 4) and *N* = 5 (day 7).

**Figure 8:**
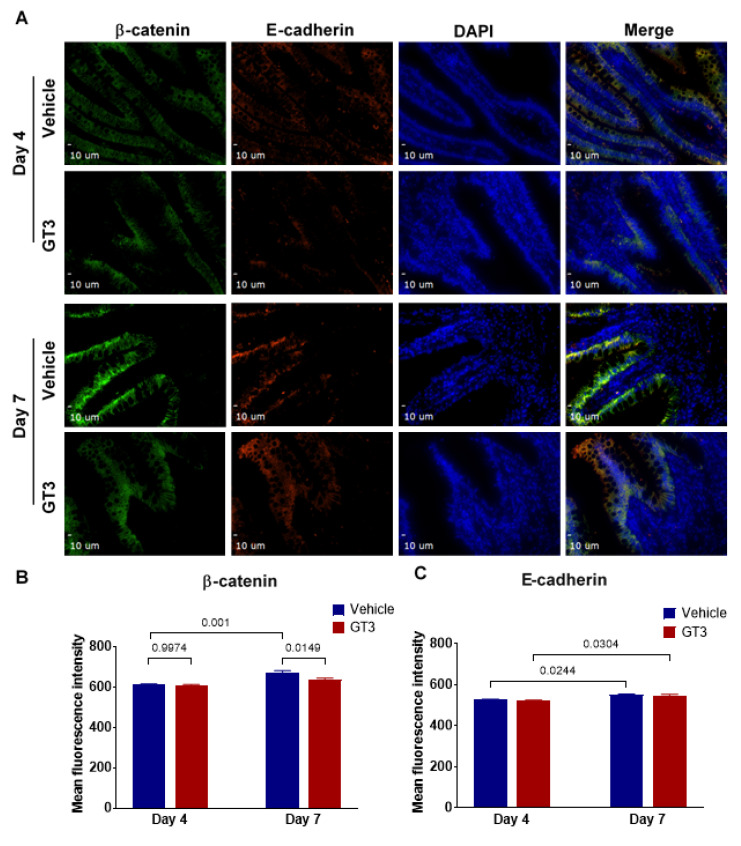
Effects of PBI on β-catenin and E-cadherin in the jejunum. (**A**) Representative photomicrograph of β-catenin expression (green) and E-cadherin expression (red) in the cross-sections of the jejunum at day 4 and day 7 post-irradiation. (**B**) Quantitation of β-catenin and (**C**) E-cadherin expression levels in the jejunum at time points indicated post-12 Gy PBI. The data are presented as average ± SEM; *N* = 3 (day 4) and *N* = 5 (day 7).

Irradiation has been shown to increase intestinal permeability at doses as low as 1–2 Gy [60]. Interestingly, we have shown GT3 to effectively reduce gut hypermeability in a mouse model exposed to TBI [61]. To evaluate if GT3 could contribute to the restoration of the epithelial barrier dysfunction induced by irradiation, here we carried out immunofluorescence staining by using a panel of TJs-related antibodies. We observed no significant difference in the expression levels of claudin-2 in vehicle- or GT3-treated groups at day 4; however, by day 7, a significant increase was noted in the GT3-treated group (*p* < 0.03), when compared to vehicle (Figure 7A,B). Interestingly, in comparison to day 4, the vehicle-treated group at day 7 showed a significant increase in β-catenin levels (*p* < 0.001), while the GT3-treated group exhibited a significant decrease at day 7 (*p* < 0.02) when compared to vehicle at day 7 (Figure 8A,B). On the contrary, E-cadherin (a major component of adherens junction) expression levels were significantly increased at day 7 in both vehicle- and GT3-treated groups (*p* < 0.02) when compared to their respective groups at day 4 (Figure 8A–C). However, no difference was seen between the groups at either day 4 or day 7. Furthermore, there were no significant differences observed at either day 4 or day 7 in the expression levels of ZO-1, claudin-7, and occludin between the vehicle- and GT3-treated groups (data not shown). In contrast, GT3 significantly increased ZO-1 levels in NHPs receiving 12 Gy TBI at day 7 [29].

## 4. Discussion 

Intentional or unintentional exposure to high doses of radiation results in GI injury—an important element of ARS. However, there exist no effective MCM/therapeutic interventions to mitigate GI damage or reduce the mortality of radiation-injured patients [11,62]. Acute GI-ARS treatment/care therefore depends on the adequate use of supportive care. The NHPs are a useful model for studies of human ARS, as they are closest to humans with respect to their genetic homology and pathophysiology [63,64,65]. We recently reported a promising role of GT3 in intestinal epithelial and crypt cells in the small intestine in an NHP model exposed to a supralethal total-body dose of 12 Gy irradiation [29]. We used the same supralethal radiation dose in the present study to study the effects of a radiation countermeasure under development. GT3 is a radiation countermeasure for H-ARS and has limited effects against a supralethal dose of radiation inducing GI-ARS. We have also conducted a transcriptomic study, and in that study there was also limited effects of GT3, but the supralethal radiation dose demonstrated significant transcriptomic changes [40]. This study was undertaken to investigate the potential of GT3 in accelerating GI recovery in NHPs receiving PBI at a dose of 12 Gy with 5% sparing of BM. 

PBI has important consequences in MCM development and radiation-induced disease progression. The hematopoietic system can recover better after high-dose irradiation if a small percentage of BM is spared/shielded. The PBI NHP model with 5% sparing of the BM used in this study was developed to mimic radiation exposures that would likely spare some BM in a radiological/nuclear event. Under such accidental or terrorist events, radiation exposure may not be total-body and uniform. The BM sparing model permits the analysis of morbidity and mortality, typically associated with acute GI-ARS, while preserving enough BM to survive through the hematopoietic syndrome [30]. With time, investigators in the field of MCM development have realized the importance of this novel model to investigate the acute and delayed effects of radiation exposure and MCM efficacy. Using a PBI model has several advantages over the TBI model. It permits investigation of the detrimental effects of radiation on a particular organ system by limiting exposure only to the organ of interest. Moreover, attenuating the effects of H-ARS facilitates the study of injury to the rest of the body’s systems. Several studies have reported this novel model, encompassing the analysis of GI injury and its usefulness for the development of MCM and identification of biomarkers [30,38]. Since damage to the BM is a non-issue in the PBI model, it is a more pertinent model to use when focusing on intestinal injury. GT3 is an encouraging MCM with significant protective efficacy both in mice and NHPs [24,27,66]. In the TBI NHP model, a single dose of GT3 with no supportive care was found to be equally effective in ameliorating hematopoietic injury as multiple doses of Neupogen/Leukine and two doses of Neulasta with complete supportive care [66]. GT3 administration in NHP was associated with a transient increase in the bioavailability of antioxidants, which may help combat radiation injury [67]. Indeed, tocotrienols confer higher protection against lipid peroxidation partly due to their greater mobility, better distribution, and enhanced interactions in the lipid bilayer [68,69]. Moreover, studies have shown that GT3 induces the expression of endogenous antioxidant enzymes including superoxide dismutase (SOD), glutathione peroxidase, and NADPH quinone oxidoreductase [70] and concentrate in endothelial cells 30–50 times more than alpha-tocopherol [71]. Importantly, the radio-prophylactic properties of GT3 depend not only on its antioxidant properties but also on its ability to accumulate in endothelial cells [71]. In addition, GT3 has also been reported to restore radiation-induced micro-RNA changes in NHP blood 24 h post-irradiation [72] and to restore the radiation-induced proteome back to baseline in the murine model [73]. Here, we report that a supralethal dose of 12 Gy PBI induced significant intestinal injury in the form of a decreased mucosal surface area and reduced villous height. We have also compared the PBI with the earlier reported TBI model work using NHPs and GT3. Notably, GT3 prophylaxis was associated with increased crypt survival on day 4 and regulated progenitor cell survival in the small intestine at early time points; however, the effects seemed to be lost by day 7. 

TBI with a certain portion of BM shielding (5 to 50%) has been shown to compromise the functional integrity of the intestine in NHPs [30,32,35,39,74]. Recently, we reported that PBI doses of 8, 11, and 14 Gy with 50% BM spared induced a dose-dependent increase in intestinal injury characterized by reduced villous height, crypt depth, and mucosal surface area by day 10 [75]. Studies have shown that 11 Gy PBI with 5% BM shielding in an NHP model induce maximum structural injury to the intestine by day 9 [38]. Likewise, similar damage has been reported by day 7 post-PBI to the small intestine in NHPs with 5% BM shielding [30]. Notably, NHPs exposed to 9.5 Gy total abdominal X-rays showed peak intestinal structural damage on day 7 that paralleled functional changes, including increased gut permeability, decreased nutrient absorption, and weight loss [74]. However, partial recovery of intestinal structure and function was observed by day 14 onwards [74]. This is consistent with what we observed in this study, where PBI with 5% BM sparing significantly reduced the mucosal surface area and villous height largely by day 7, in both vehicle- and GT3-treated groups, thus suggesting that mucosal integrity of the intestine was compromised. Notably, crypt depth significantly increased on day 7 in both vehicle- and GT3-treated groups, suggesting that sparing of bone marrow might have contributed to more stem cells survival and proliferation after PBI. In contrast, in NHPs receiving 12 Gy TBI, it was only the GT3-treated group that was associated with enhanced crypt depth at day 7 [29]. Moreover, studies have shown a strong positive correlation between crypt depth and stem cell survival following irradiation [53]. 

The renewal of intestinal epithelium relies on stem cells located at the bottom of the crypt [56,59,76]. In addition, the intestinal epithelium not only has the ability to constantly self-renew but also possess a tremendous capacity to repair following damage. However, increased apoptosis at high radiation doses may fail to trigger a regenerative response, leading to GI failure [57]. Two critical determinants of the extent of jejunal damage are apoptotic cell death and loss of crypt cell proliferation [77]. Importantly, we have shown jejunum as one of the most radiosensitive regions of the small intestine [75]. Interestingly, in this study, GT3 significantly enhanced crypt survival at day 4 post-PBI; however, the effect was lost by day 7. Surprisingly, GT3 did not seem to influence cell death in the small intestine but showed increased progenitor cell survival at an early time point, though the level of significance was not achieved. This is in contrast to what we observed in NHPs receiving 12 Gy TBI, where GT3 significantly reduced intestinal cell death in jejunum and enhanced cell proliferation at day 7 post-radiation [29]. Indeed, studies have shown a significant difference in biological outcome using gamma ray irradiation versus X-ray irradiation. Moreover, GT3 protects endothelial cells from radiation-induced injury not only because of its antioxidant properties but also by inhibition of HMG-CoA reductase and by enhancing the availability of nitric oxide synthase co-factor tetrahydrobiopterin [78]. Both GT3 and tetrahydrobiopterin supplementation have been shown to reduce post-irradiation vascular peroxynitrite production [78]. However, in our current model, GT3 exhibited minimal protective effect, unlike what we observed in our TBI model [29]. It is conceivable that by changing the geometry from a total to a partial-body irradiation, the actual dose absorbed by the GI-tract may be higher than the given radiation dose. Though there was 5% bone marrow sparing in PBI, these cells may not be of much help against such a supralethal dose of radiation inducing GI-ARS. In addition, the dose rate in the two studies was different: 0.6 Gy/min in TBI and 1.4 Gy/min in PBI. We had to use two different radiation sources for PBI and TBI for specific reasons. LINAC is preferred for PBI, as it provides a collimated beam, and the radiation field is limited. Therefore, one can place only the part of the body in the field that one is interested in exposing. The high-level cobalt facility used for gamma irradiation has a panoramic field, and it is not possible to provide complete sparing of any body part one is interested to spare.

Citrulline, a nonprotein amino acid, is produced by the enterocytes of the small intestine [79] and has been characterized as a biomarker for radiation injury associated with GI-ARS [38,54,55]. An excellent correlation between markers of GI injury and plasma citrulline levels is well-established [37,42,43,44,80]. Importantly, lower levels of citrulline reflect the present status of the functional enterocytes [81]. Moreover, a single TBI dose of 1–12 Gy showed a significant, radiation dose-dependent reduction in citrulline levels at 3.5 to 4 days post-irradiation [43]. Consistent to this, we have reported that exposure to 6.7 Gy and 7.4 Gy total-body radiation (mainly associated with the H-ARS) in the NHP model resulted in significant dose-dependent reductions in plasma citrulline levels by day 4 [82]. By day 12, citrulline levels slightly recovered but did not reach to normal levels in either dose of radiation. Likewise, reduced citrulline levels were seen by day 4 in NHPs exposed to radiation doses of 7.5 to 13.0 Gy TBI as well as 11.0 Gy PBI with 5% BM spared [38]. Interestingly, a steady increase in plasma citrulline was observed by day 15 onwards coupled with intestinal recovery in NHPs receiving 11 Gy PBI with 5% BM shielding. Here, we report that exposure to 12 Gy partial-body radiation resulted in a significant decrease in plasma citrulline levels in both vehicle- and GT3-treated groups by day 4, which lasted until day 7 (latest time point examined in this study), thus suggesting decreased intestinal epithelial cell mass. These findings are consistent with what we have observed in our other studies of intestinal injury. GT3 did not seem to ameliorate radiation-induced decrease in plasma citrulline levels in NHPs exposed to a supralethal X-ray dose of radiation. 

Ionizing radiation causes structural and functional changes to the GI tract, including increased gut permeability through alteration in epithelial tight junctions (TJs) [74,83]. Intestinal barrier integrity is maintained by the highly specialized intercellular junctional complexes such as TJs, adherens junctions, and desmosomes [84]. These TJs are mainly composed of transmembrane proteins such as occludins, claudins, and junctional adhesion molecules, which interact with the zona occludens, intracellular adapter proteins [85], and are disrupted following irradiation. We have recently shown that TBI negatively affects TJs-related proteins in the NHP model, which are crucial for maintaining mucosal integrity [83,86]. Furthermore, mucosal barrier impairment was observed in NHPs at doses ranging from 10–12.5 Gy TBI with 5% BM shielding in both the small intestine and colon, where the changes in the small intestine preceded those in the colon [30,32]. In the current study, GT3 modulated the expression of claudin-2 and β-catenin on day 7 post-PBI. Notably, claudin-2 is highly induced in leaky epithelial tissues, including crypts [87]. Upregulation of E-cadherin was noted at day 7 in both the GT3- and vehicle-treated groups. Importantly, E-cadherin is not only critical for intestinal homeostasis but is also important for maturation of Paneth and goblet cells [88]. Interestingly, GT3 showed a trend of increased ZO-1, claudin-7, and occludin levels at day 4, but the trend did not persist by day 7 (data not shown). Taken together, these data suggest that 5% bone marrow sparing might not be sufficient to ameliorate radiation-induced increase in gut permeability. In addition, extensive intestinal damage inflicted by X-rays at a dose of 12 Gy PBI cannot be ruled out. 

In summary, GT3 was associated with increased crypt survival at day 4 and exhibited the potential to regulate progenitor cell survival in the small intestine at an early time point. However, the effects seemed to be lost by day 7. To our knowledge, this is the first report with GT3 using the partial-body NHP radiation exposure model with LINAC (X-ray source). These data suggest that X-rays induce substantial intestinal injury compared to gamma rays at early time points. Future studies are clearly warranted to compare GT3 effects using X-rays and gamma rays with increased N, necessitating radiation dose reduction to achieve comparable biological effects. These findings may have therapeutic implications for the development of MCM.

## Figures and Tables

**Table 1:** Experimental design for 16 NHPs exposed to 12 Gy PBI.

Study Design with 16 NHPs					
GI Study (Partial-Body Irradiation, LINAC, 1.4 Gy/min)					
NHP	Drug	Route	Dose	Frequency	Irradiation Dose (Gy)
8 (4 M/4 F)	GT3	Sc	37.5 mg/kg	24 h prior to irradiation	12
8 (4 M/4 F)	Veh	Sc	37.5 mg/kg	24 h prior to irradiation	12

**Table 2:** Euthanasia schedule for 16 NHPs exposed to 12 Gy PBI.

Euthanasia Schedule for GI Injury		
Groups	Day 4 Post-Irradiation	Day 7 Post-Irradiation
GT3 + 12 Gy	3	5
Vehicle + 12 Gy	3	5

## Data Availability

All relevant data, which support the findings of the study, are within the manuscript.
